# Molecular modeling, docking and protein-protein interaction analysis of MAPK signalling cascade involved in Camalexin biosynthesis in Brassica rapa

**DOI:** 10.6026/97320630014145

**Published:** 2018-04-30

**Authors:** Manu Gaur, Apoorv Tiwari, Ravendra P Chauhan, Dinesh Pandey, Anil Kumar

**Affiliations:** 1 Department of Molecular Biology and Genetic Engineering, College of Basic Sciences and Humanities, G.B. Pant University of Agriculture and Technology, Pantnagar 263145, Uttarakhand, India; 2 Sam Higginbottom University of Agriculture, Technology & Sciences, Allahabad 211007, Uttar Pradesh, India

**Keywords:** MAPK, MKK, Camalexin, Botrytis cinerea, Brassica rapa, Alternaria brassicae

## Abstract

Phytoalexins are small antimicrobial molecules synthesized and accumulated by plants upon exposure to pathogens. Camalexin is an indole-derived phytoalexin, which is accumulated in plants including Arabidopsis thaliana, and other Brassicaceae, which plays a major role in disease resistance against fungal pathogens. The productivity of Brassica crops is adversely affected by Alternaria blight disease, which is caused by Alternaria brassicae. In Arabidopsis thaliana, MAP kinase signalling cascade is known to be involved in synthesis of camalexin, which contributes to disease resistance against a necrtrophic fungal pathogen, Botrytis cinerea. In the present study, MAPK signalling cascade leading to biosynthesis of camalexin that triggers defense responses in B. rapa upon exposure to the most devastating nectrophic fungus, Alternaria brassicae has been elucidated with the help of previously reported MAPK cascade in Arabidopsis thaliana, Molecular modelling, docking, and protein-protein interaction analysis of MAP kinases retrieved from Brassica rapa genome have been carried out to reveal the above cascade. The tertiary structure prediction of MAPKs obtained through molecular modelling revealed that all the protein models fulfil the criteria of being the stable structures. The molecular docking of predicted models for elucidating potential partners of MAPKs revealed strong interactions between MKK1, MKK4, MKK5, MAPK3 and MAPK6 with MKK9. The MAPK signalling cascade also shows different genes that express and play major role in camalexin biosynthesis in B. rapa during defense response to A. brassicae. The understanding of MAPK defense signaling pathway in B. rapa against devastating fungal pathogen Alternaria brassicae would help in devising strategies to develop disease resistance in Brassica crops.

## Background

The host-pathogen interaction is precisely driven by selection to acquire improved defense against pathogens, therefore, during the course of evolution, plants have adapted to the processes that trigger the synthesis and accumulation of a vast array of structurally diverse anti-microbial secondary metabolites. Diversity among the secondary metabolites is considered to be partly originating from this host-pathogen interaction as a byproduct of the evolutionary process [1]. This notion reflects that each particular class of secondary metabolites is restricted to a narrow phylogenetic lineage, which is responsible for developing disease resistance against specific pathogens. Among these antimicrobial secondary metabolites, phytoalexins represent an important class which can be defined as small molecular weight anti-microbial compounds that are synthesized de novo by plants in response to an attack and progression of the fungal pathogens and are considered to be the major determinants of induced plant resistance against these pathogens [2]. However, the regulatory mechanisms for phytoalexin biosynthesis is still largely unknown but, previous investigations have suggested that the mitogen activated protein kinase (MAPK) signalling pathways are the core of the defense mechanisms that regulate phytoalexin biosynthesis, often orchestrated in microbe-associated molecular patterns (MAMP) triggered immunity [3]. In a recent study, it was found that the MAPK cascade activate transcription factors to regulate the phytoalexin biosynthesis in MAMP triggered immunity [4]. MAMP-triggered immunity exerts a number of defense responses upon recognition of reactive oxygen species (ROS) accumulation including the synthesis of anti-microbial secondary metabolites or phytoalexins alongwith transcriptional activation of pathogenesis-related genes [5]. The MAMP-signal is received by plant pattern recognition receptors (PRRs) that are shortly followed by the activation of plant MAPK signaling pathways consisting of three kinase-signaling modules (MAPK, MAPK kinase (MAPKK), and MAPKK Kinase (MAPKKK)) [3].

Recent investigations have revealed that there is a typical indolederived phytoalexin known as 'Camalexin' that is synthesized and accumulated in Arabidopsis thaliana to trigger defense against a fungal pathogen Botrytis cinerea [6]. Infection to Arabidopsis thaliana with Botrytis cinerea is recognized by the pattern recognition receptors (PRRs) of the host which then express the genes involved in the downstream processing of the MAPK signalling cascade to synthesize and accumulate camalexin in order to trigger defense against the pathogen [6]. It has previously been reported that the biosynthesis of camalexin is regulated by MAPK signalling pathway via expression of AtMPK3, AtMPK4 and AtMPK6 promoters in Arabidopsis thaliana [7]. Interestingly, a novel MAPK signalling pathway is recently reported in rice where, OsMPK4, and OsMPK3/OsMPK3 genes induce the production of diterpenoid phytoalexins [8]. AtWRKY33, which is a DNA binding protein [7] and OsTGAP1, which is a bZIP transcription factor, [8] have both been identified to be the inducers of diterpenoid phytoalexin biosynthesis in Arabidopsis and rice, respectively.

The production of Brassica crops, which are economically important oil-seed, crops in India and worldwide is compromised due to fungal diseases. Apart from the necrotrophic fungal pathogen Botrytis cinerea [9], the Alternaria brassicae is another major necrotrophic fungal pathogen [10] that causes significant yield losses in Brassica rapa and other Brasicaceae [10]. In Brassica crops, Alternaria brassicae incites Alternaria blight disease against which little information is available on the defense pathways. Fortunately Arabidopsis thaliana has been demonstrated as host for Alternaria brassicae (6), and the genes of MAPK signaling cascade in Arabidopsis thaliana are evolutionary conserved [6, 7]. Therefore, in the present study, MAPK signaling cascade responsible for biosynthesis of camalexin in Brassica rapa has been elucidated using the genomic information from Arabidopsis. The above defense signaling pathway triggered in response to Alternaria brassicae can further be validated through in vitro experiments. The understanding of MAPK defense signaling pathway in Brassica rapa against Alternaria brassicae pathogen would help in formulating strategies to develop resistance in Brassicacrops against Alternaria blight.

## Methodology

### Sequence retrieval

Different MAPKs were retrieved from Brassica rapa genome database available online at http://brassicadb.org/brad/ by searching homologous sequences of MAPK of Arabidopsis thaliana.

### Structure prediction

Protein sequences of MAPKs were retrieved from the B. rapa genome database and the RaptorX server was used for three dimensional structure prediction (http://raptorx.uchicago.edu/StructurePrediction/predict/).

### Molecular docking

ClusPro server (https://cluspro.org) was used for protein-protein docking [11] of different MAPKs and the scores were recorded in the form of lower energy expressed in Kcal/mol. The ClusPro server is a widely used tool for protein- protein-docking analysis. This server provides a simple homepage for basic use, requiring only two files in Protein Data Bank (PDB) format. Docking study of the chemical molecule with the proteins for camalexin biosynthesis was performed using PatchDock server [12, 13]. Chemical molecules were treated as ligand and proteins were treated as receptor molecules. The PatchDock algorithm is inspired by the object recognition and image segmentation techniques. The algorithm has the following three major stages:

#### I. Molecular shape representation

Firstly, computing the molecular surface of the molecules. Then, a segmentation algorithm was applied for detection of geometric patches (concave, convex and flat surface pieces). The patches were filtered so that the patches only with 'hot spot' residues were retained.

#### II. Surface patch matching

The patches detected in the molecular shape representation were matched with application of a hybrid of the Geometric Hashing and Pose-Clustering matching techniques. The concave patches were matched with convex patches, and the flat patches were matched with the possibility of any type of patches that may be present.

#### III. Filtering and scoring

The candidate complexes generated from the surface patch matching were examined. We discarded all complexes with unacceptable penetrations of the atoms of the receptor to the atoms of the ligand. Finally, the remaining candidates were ranked according to a geometric shape complementarity score.

### Superimposition of predicted 3D structures

SuperPose tool [14] was used for superimposing the structure of different kinases. SuperPose is a serverthat calculates protein superimposition using a modified quaternion approach. From a superposition of two or more structures, SuperPose generates sequence alignments, structure alignments, PDB coordinates, RMSD statistics, Difference Distance Plots, and interactive images of the superimposed structures. The SuperPose web server supports the submission of either PDB-formatted files or PDB accession numbers.

### Protein-protein interaction

Protein-protein interaction was predicted by the STRING database [15]. The STRING database contains information from numerous sources, including experimental data, computational prediction methods and public text collections. STRING database also serves to highlight functional enrichments in user-provided lists of proteins, using a number of functional classification systems such as GO, Pfam and KEGG. Interestingly, the latest version 10.0 contains information about 9.6 million proteins from more than 2000 organisms.

### Pathway for camalexin biosynthesis

CellDesigner tool [16] was used to construct the pathway by using Plant Cyc database and different research resources as a reference. CellDesigner was used to draw the pathway. CellDesigner is a structured diagram editor for drawing gene-regulatory and biochemical networks.

## Results and Discussion

### Sequence retrieval of Different MAPKs involved in phytoalexin biosynthesis in Brassica rapa

MAPK cascade is a conserved signalling cascade that triggers defense against a vast array of pathogens. In the case of MAPK signalling cascade in Arabidopsis thaliana against Botrytis cinerea, MKK1, MAPK3, MKK4, MKK5, MAPK6 and MAPK9 are actively involved. The Nucleotide sequences of these Kinases were retrieved by BLASTn search against Brassica rapa genome database using MAPK of Arabidopsis thaliana as query. The protein sequences of these kinases were also retrieved. The BLASTn algorithm showed significant similarity between MAPK sequences of Arabidopsis thaliana and Brassica rapa. This indicates towards the conserved nature of the MAPK sequences among plant species. Sequences with maximum identity and lowest Evalue were considered for homology modelling.

### Homology modelling and molecular docking of MAPK

**Figure 1:**
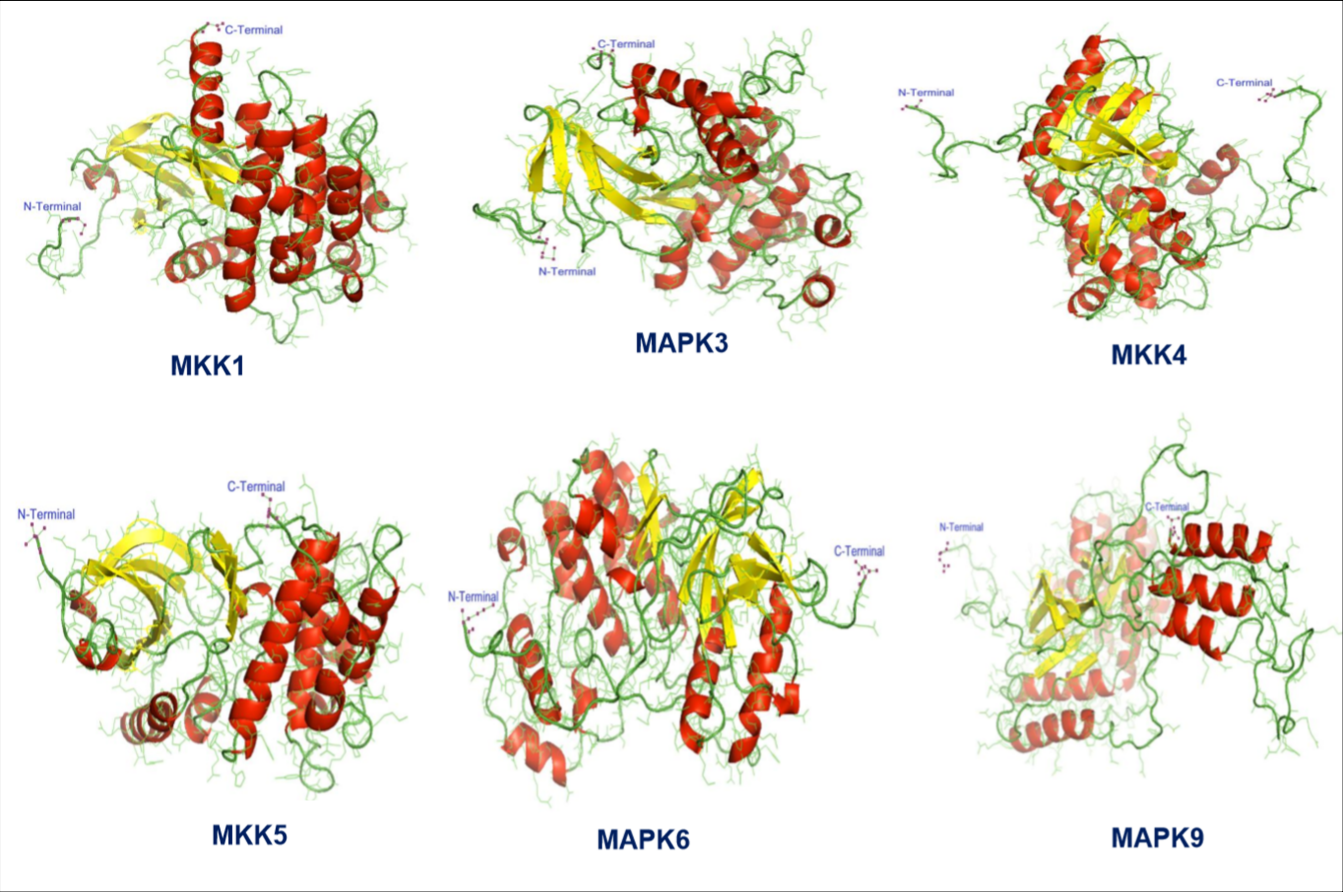
Illustration of tertiary structures of MAPKs involved in biosynthesis of the indole-derived phytoalexin camalexin in Brassica rapa against A.brassicae using RaptorX server.

Tertiary structure prediction of MAPKs involved in biosynthesis of the indole-derived phytoalexin camalexin in Brassica rapa was carried out by RaptorX server (Figure 1), that was followed by the validation of the protein structures produced by computational modelling approach. Interestingly, all the structures were observed to follow the criteria for being the stable structure given that the criteria for the expected percentage of number of residues allowed in favoured region and allowed region be ~98.0% and ~2.0%, respectively.

Previous studies have considered the stability of the structures having the value of the favoured regions >90 percent [17]. In this analysis, the protein models of MAPKs and MKKs of Brassica rapa consistently showed the percentages of the favoured regions above 90%, therefore, they are predicted to be having stable 3D structures. All the MAPK models have been submitted to the protein model database (PMDB) with accession numbers [18].

**Figure 2:**
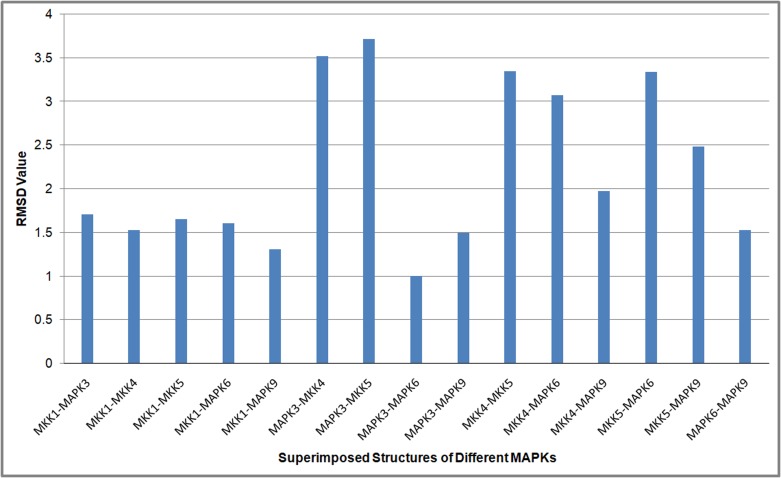
Superimposition of the predicted model performed by Superpose server. Root mean square deviation (RMSD) values predict the structural variation between 3D protein structures; lower RMSD value reflects higher structural similarity and, vice versa.

**Figure 3:**
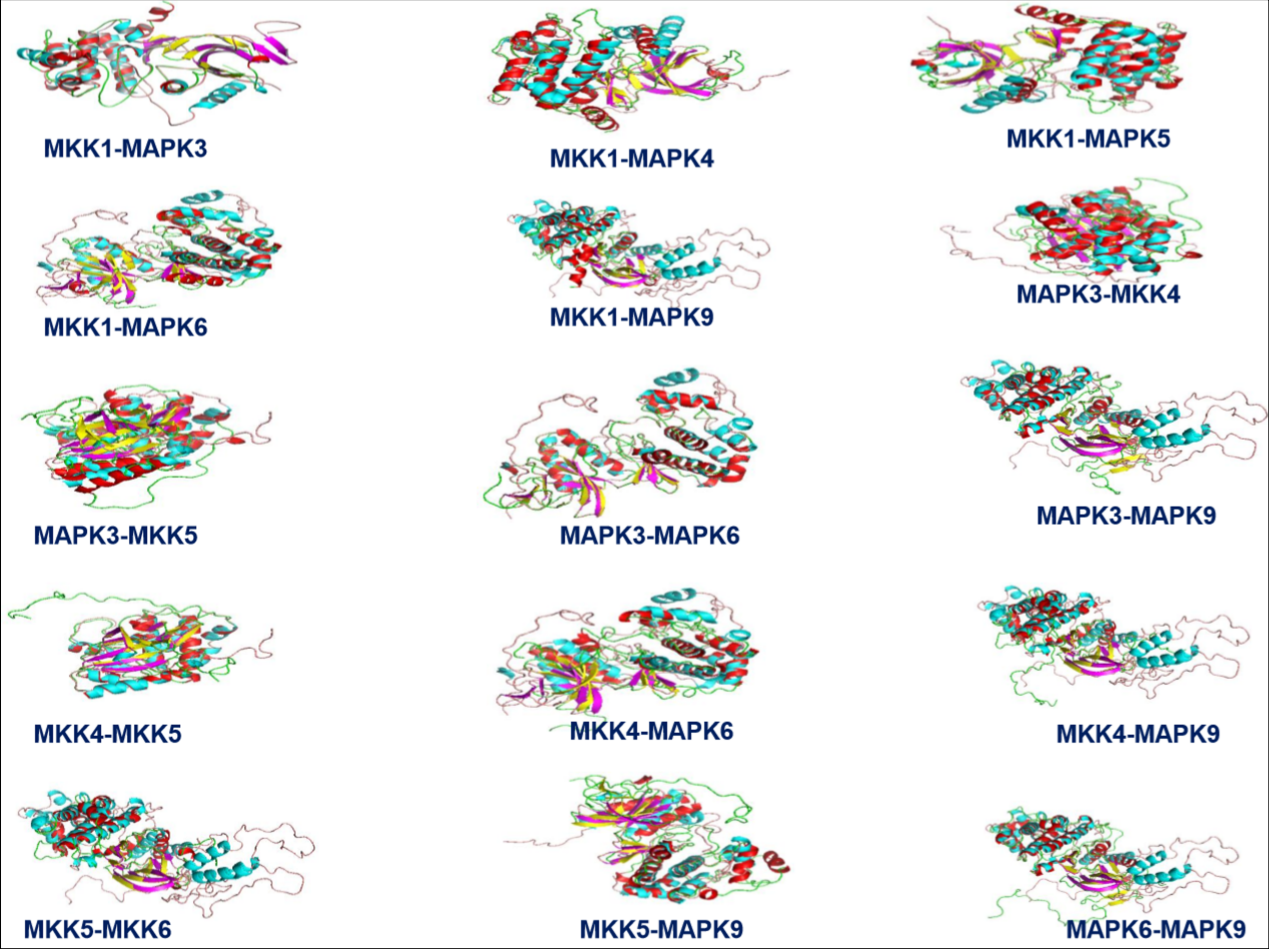
Superimposition of tertiary structures of MAPKs involved in camalexin biosynthesis in Brassica rapa against A.brassicae using Pymol software.

Root mean square deviation (RMSD) values predict the structural variation between 3D protein structures (Figure 2); lower RMSD value reflects higher structural similarity and, vice versa. MAPK3-6, MKK1-MAPK9, MAPK3-9, MKK1-4, MAPK6-9 showed the highest similarities at structure level given lower RMSD values (>1.5). Apart from this, MKK1-MAPK6, MKK1-5, MKK1-MAPK3, MKK4-MAPK9, and MKK5-MAPK9 were found moderately similar given the RMSD values between 1.5 and 3.0, while MKK4-MAPK6, MKK5-MAPK6, MKK4-5, MAPK3-MKK4 and MAPK3-5 having RMSD values in the range of 3 to 3.7 showed relatively less similar structure. The structural similarity between particular proteins is considered a good indicator of functional similarity because the amino acid sequences of the protein determine their 3D structures, which in turn, determine their functional properties [19]. This was further confirmed with the help of superimposition of tertiary structures of MAPKs involved in camalexin biosynthesis in Brassica rapa against A.brassicae using Pymol software and it was observed that the RMSD values of MAPKs were supported for the structural similarities (Figure 3).

### Pathway for phytoalexin synthesis in Brassica rapa

**Figure 4:**
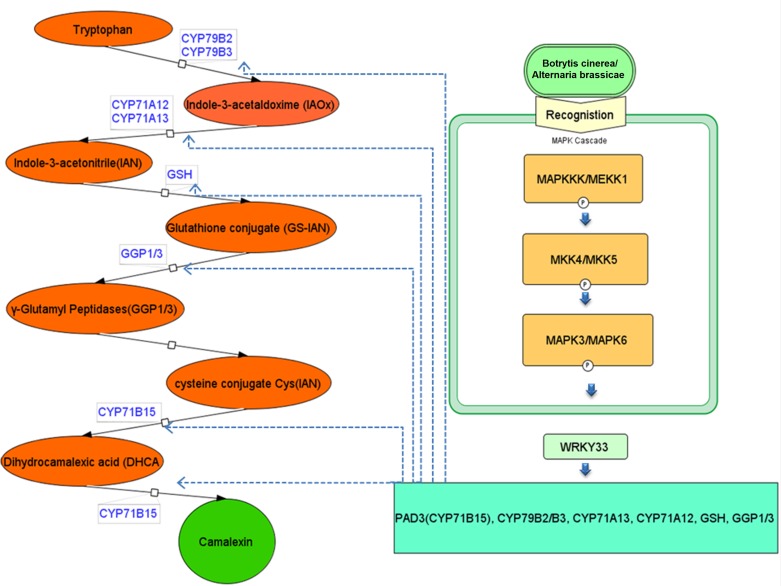
Camalexin biosynthesis pathway and regulatory gene expressed by MAPK cascade designed using cell designer tool.

Upregulation of the specific genes (CYP71A12, CYP71A13, and CYP71B15) alongwith upstream Trp biosynthetic genes and CYP79B2 is essential for the biosynthesis of camalexin [8]. The known regulator of camalexin, WRKY33, binds to the promoters of CYP71B15 and CYP71A13 [20]. The pathway for biosynthesis of camalexin comprises two major steps; in the first step, the fungus Botrytis cinarea attacks the Brassica rapa and the recognition system gets activated. Upon activation of the recognition system the MAPK cascade becomes functional where MAPKKK/MEKK1 phosphorylates into MKK4/MKK5, which further gets phosphorylated into MAPK3/MAPK6. WRKY33 is a molecular target of the MPK3/6 cascade; WRKY33 binds to the promoter of CYP71B15 [21]. The pathway is depicted in Figure 4, which was designed by CellDesigner software with existing information. By this cascade different genes get expressed and play a major role in the biosynthesis of camalexin (Figure 4).

### Molecular docking

ClusPro docking server for predicting potential partners of MAPK carried out molecular docking of predicted models. Protein-protein docking ClusPro server provides the results in four manners i.e. Balanced, Electrostaticfavored, Hydrophobic-favored and VdW+Elec, where the energy is calculated in the form of coefficient wattage by using the formula E=0.40Erep+-0.40Eatt+600Eelec+1.00EDARS in the Balanced manner [11].

The results of docking were stored in the form of different clusters; cluster '0' has lowest energy among all the clusters, therefore, for scoring the docking results, the cluster '0' was selected. It is important to note that the lower energy in the form of negative energy score reflects higher affinity. The MKK4- MAPK9 has the lowest energy, which means that the interaction between MKK4 and MAPK9 is more stable than between other MAPKs (Table 1).

**Figure 5:**
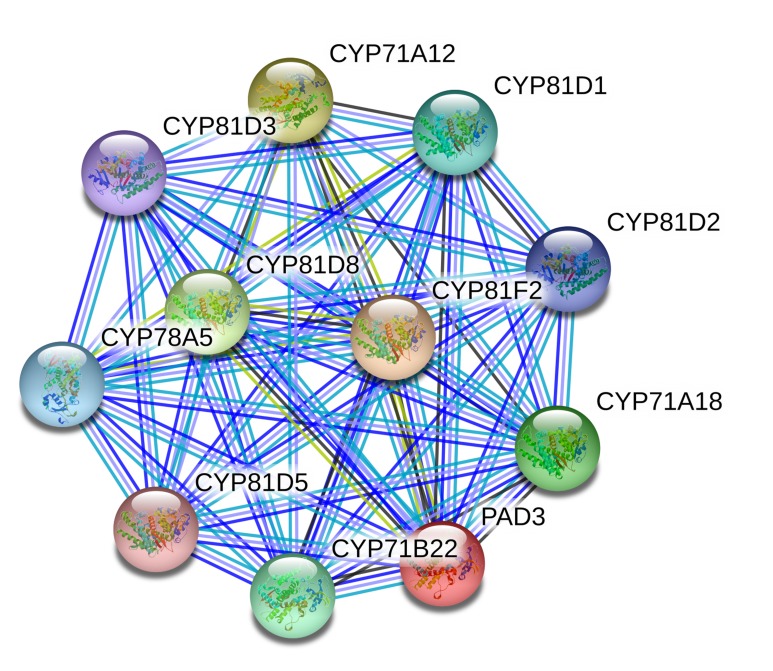
Protein-protein interaction network of PAD3 protein with the other associated proteins involved in the biosynthesis of the indole-derived phytoalexin camalexin. Catalyzes two reactions, the formation of dihydrocamalexate from indole-3-acetonitrilecysteine conjugate and the oxidative decarboxylation of dihydrocamalexate that is the final step in camalexin biosynthesis. Required for the resistance to the fungal pathogens, B.cinerea, A. brassicae. The network reveals CYP71A12 is the potential functional partner of PAD3. Protein-protein interaction was predicted by the STRING database.

The MAPK signalling cascade activates different genes involved in camalexin biosynthesis, therefore, it is essential to study each step of the pathway regulation, which can be achieved, by studying protein-ligand interactions. This was done as depicted in Table 2. It was observed that CYP79B2 and CYP79B3 docked with tryptophan with binding energy of -86.70, -204.52 kcal/mol, respectively. This illustrates that CYP79B3 is a good interacting partner of tryptophan given lower energy. Further energy scores were calculated for other proteins that showed probable interacting partners (Figure 5; Table 2). This data can further be confirmed with in vitro mutant analyses. Mutations in pad3 are defective in biosynthesis of the indole-derived phytoalexin, camalexin. PAD3 encodes a cytochrome P450 enzyme that catalyzes the conversion of dihydrocamalexic acid to camalexin. Multifunctional enzyme involved in the biosynthesis of the indole-derived phytoalexin camalexin catalyzes two reactions; the formation of dihydrocamalexate from indole-3-acetonitrilecysteine conjugate and the oxidative decarboxylation of dihydrocamalexate that is the final step in camalexin biosynthesis.

Here, scores given in Table 2 represent the geometric shape complementarity scores; the solutions are sorted according to this score. Area represents approximate interface area of the complex. ACE represents the atomic contact energy. The CYP79B2 converts tryptophan to indole-3-acetaldoxime, a precursor for tryptophanderived glucosinolates, and indole-3-acetic acid (IAA) [22, 23] is involved in the biosynthetic pathway to 4-hydroxyindole-3- carbonyl nitrile (4-OH-ICN), a cyanogenic metabolite required for inducible pathogen defense [24]. GGP1 is involved in glucosinolate biosynthesis. GGP1 hydrolyzes the gammaglutamyl peptide bond of several glutathione (GSH) conjugates to produce Cys-Gly conjugates related to glucosinolates. The gamma-Glu-Cys-Gly-GSH conjugates are the sulfur-donating molecule in glucosinolate biosynthesis [25, 26]. GGP1 converts phenylacetohydroximoyl-GSH to benzylglucosinolate [25]. GGP1 can use the GSH conjugate of the camalexin intermediate IAN (GS-IAN) as substrate which is required for the biosynthesis of camalexin, a pathogen-inducible phytoalexin with antibacterial and antifungal properties [26].

The functional proteins association networks analysis used STRING database version 10.5 where all the proteins involved in biosynthesis of camalexin in Brassica rapa were considered for interaction prediction. Figure 5 depicts the interacting network where PAD3 was observed as the major functional node with multiple protein interactions. In the MAPK signalling cascade, different genes get expressed and play a major role in the biosynthesis of camalexin in Brassica rapa. The understanding of MAPK defense signaling pathway in Brassica rapa against fungal pathogen Alternaria brassicae would help in devising strategies to develop disease resistance in economically important oil-seed crop Brassica rapa.

## Conclusions:

Recognition of pathogen with the help of pattern recognition receptors of the host trigger a downstream cascade of MAPK signalling to provide resistance to the host against pathogen attack. MAPK is a conserved signalling cascade and we observed a significant similarity between MAPK sequences of Arabidopsis thaliana and Brassica rapa which further supports the conserved nature of the MAPK sequences. In MAPK signalling cascade of Botrytis cinerea different MKKs and MAPKs were found to be actively involved. Highest similarities at structure level between MAPK3-MAPK6, MKK1-MAPK9, MAPK3-MAPK9, MKK1- MKK4, MAPK6-MAPK9 proteins followed by moderate similarities between MKK1-MAPK6, MKK1-MKK5, MKK1- MAPK3, MKK4-MAPK9, and MKK5-MAPK9 proteins alongwith fairly less similarities between MKK4-MAPK6, MKK5-MAPK6, MKK4-5, MAPK3-MKK4, MAPK3-5 MAPK proteins proved good functional similarities between MAPK proteins given The fact that structural similarity between particular proteins is considered a good indicator of functional similarity. The multifunctional enzyme involved in the biosynthesis of camalexin is involved in the formation of dihydrocamalexate from indole-3-acetonitrile-cysteine conjugate and the oxidative decarboxylation of dihydrocamalexate, which is the final step in the camalexin biosynthesis. This study is the first attempt to unravel the MAPKs defence signalling cascade in Brassica rapa against Alternaria brassicae by utilizing the knowledge on MAPK signalling cascade triggered against Botrytis cinerea in Arabidopsis thaliana. The reported in silico findings would be helpful for understanding the plant defence mechanisms of Brassica crops against Alternaria brassicae pathogen which will further help in developing disease resistance in these economically important oil seed crops.

## Figures and Tables

**Table 1:** Molecular docking of MAPK partners

S.No.	Docking Complex	Energy
1	MKK4- MAPK9	-1320
2	MAPK6- MAPK9	-1257
3	MKK1- MAPK9	-1178
4	MAPK3- MKK4	-1166
5	MAPK3- MAPK9	-1107
6	MKK5- MAPK9	-1106
7	MAPK3- MAPK6	-1070
8	MKK4- MKK5	-1068
9	MKK1- MAPK3	-1066
10	MAPK3- MKK5	-1037
11	MKK1- MKK4	-995.6
12	MKK1- MKK5	-943.3
13	MKK1- MAPK6	-938.5
14	MKK4- MAPK6	-931
15	MKK5- MAPK6	-857

**Table 2:** Docking study of camalexin biosynthesis pathway; proteins and their interacting partners

S.No.	Docked Molecule	Score	Area	ACE (kcal/mol)
1	Tryptophan_CYP79B2	3512	424.70	-86.7
2	Tryptophan_CYP79B3	3898	441	-204.52
3	Indole3acetaldoxime_CYP71A13	3184	367.3	-11.47
4	Glutathion_Indole3acetonitrile	1444	154.1	-108.89
5	GGP1_IANglutathioneconjugate	5670	682.9	-284.35
6	Cys(IAN)_PAD3	4750	591.5	-337.49
7	DHCA_PAD3	3990	509.5	-306.13
